# Characterization of *Alternaria alternata* and *Alternaria scrophulariae* Brown Spot in *Colombian quinoa* (*Chenopodium quinoa*)

**DOI:** 10.3390/jof9090947

**Published:** 2023-09-20

**Authors:** Ingrid Rocío Fonseca-Guerra, Mayra Eleonora Beltrán Pineda, Martha Elizabeth Benavides Rozo

**Affiliations:** Enviromental Management Investigation Group, Universidad de Boyacá, Tunja 150003, Colombia; mebeltran@uniboyaca.edu.co (M.E.B.P.); biomeb2260@gmail.com (M.E.B.R.)

**Keywords:** quinoa, *Alternaria*, pathogenicity, brown spot

## Abstract

*Alternaria* is a saprophytic and opportunistic fungus with a worldwide distribution that can affect the quality of various agricultural products, such as fruits, cereals, and pseudocereals. This research was carried out to investigate the population of this genus associated with quinoa cultivation in plots located in the Boyacá department (Colombia), the country’s third-largest quinoa-producing department. The present study found 17 *Alternaria* isolates, of which 13 were identified as *A. alternata* and 4 as *A. scrophulariae* (formerly *A. conjuncta*) employed molecular markers of internal transcribed spacer (ITS) region and translation elongation factor 1α (TEF-1α). In the pathogenicity test under greenhouse conditions, all the *Alternaria* isolates showed some degree of pathogenicity on Piartal quinoa cultivar plants although no significant differences were found in isolates. The severity indices ranged from 2 to 5, and the percentage of affected leaves per plant ranged between 15% and 40%. This fungus affected the foliar tissue of quinoa, resulting in chlorotic and necrotic spots, symptoms that can generate a reduction in the quality and productivity of crops. This is the first time that the pathogenicity of *Alternaria* spp. in the Piartal variety has been described and the first report of this genera in quinoa crops of Colombia.

## 1. Introduction

*Chenopodium quinoa* is a pseudocereal that is native to South America, where it was domesticated, presumably 7000 years ago, in the Andean region near Lake Titicaca. The principal producer countries in the region are Bolivia, Peru, Ecuador, Argentina, Chile, and Colombia. The cultivation area for quinoa is approximately 250 hectares, with significant cultivation in Nariño, Cauca, Cundinamarca, and Boyacá departments, yielding an annual production of 375 tons [1]. Quinoa grains are not only rich in fiber, lipids, carbohydrates, vitamins, flavonoids, and minerals such as calcium, magnesium, and iron, but they are also gluten-free, possess a low glycemic index, and contain a balanced composition of nine essential amino acids, with notable concentrations of histidine, lysine, and methionine [2]. This pseudocereal offers high nutritional value and agronomic versatility, making it pivotal for enhancing food security, particularly in regions where protein sources are limited and food production necessitates reliance on imports.

Although quinoa possesses varieties that exhibit resistance to various biotic and abiotic factors, several diseases affecting quinoa plants have been documented. These diseases are caused by bacteria, viruses, oomycetes, and fungi that primarily target the stems and leaves of plants [3]. Diseases attributed to oomycetes and fungi comprise mildew and damping-off, which are induced by *Peronospora farinosa* and *P. variabilis* [4,5], as well as *Fusarium* spp. and *Pythium zingiberum* [6]. Additionally, there are instances of leaf spots and panicle disease caused by *Alternaria*. This disease manifests during the grain-filling stage, displaying symptoms such as the emergence of a pale pink, grayish white, or dark brown moldy film on the grains. These symptoms lead to discoloration, inadequate filling, and the deformation of the grains. The causative agents of this ailment include *Trichothecium roseum*, *A. alternata*, and *F. citri* [5]. Moreover, there exists a yellow leaf blotch disease brought about by *Alternaria* section *Alternata* and *Alternaria* section *Infectoriae* [7]. This fungus is primarily recognized as a foliar pathogen that can lead to a reduction in photosynthetic potential, albeit resulting in a low level of tissue destruction [8]. This genus holds the tenth position among phytopathogenic fungi due to its capability to infect a wide array of host plants, exceeding 400 reported susceptible species [8].

*Alternaria* belongs to the genus of ascomycete fungi within the subkingdom Eumycotera. It falls under the family Dematiaceae, the order Moniliales, and the class Hyphomycetes. The species of this genus are omnipresent within the environment, serving as saprophytes in decomposing soils while also functioning as plant pathogens. They have the capacity to displace cereal crops and induce stem and leaf spot diseases, ultimately resulting in the deterioration of fruits or grains, particularly in the postharvest phase [9].

The identification of species within this genus is intricate, particularly because a sexual phase has not been determined in several species. This absence of a sexual phase has led to the classification of this taxonomic group within the division of mitosporic fungi or the imperfect fungi Phylum. Prior to the advent of molecular techniques, the classification of these species relied on morphological characteristics observed under standard culture conditions, encompassing traits such as colony appearance, conidiogenesis, and conidia melanin production [10]. In the case of certain *A. alternata* strains, despite morphological similarities, distinct pathological variances have led to the categorization of these strains as separate *formae speciales* or pathotypes [10]. These terms have been employed to designate isolates that exhibit indistinguishable morphologies but target specific hosts. Roughly 16 *formae speciales* have been characterized for *Alternaria* strains, each sharing comparable morphological traits but differing in the production of selective host toxins. This has resulted in the identification of seven pathotypes within *A. alternata* [11]. While *A. alternata*, *A. infectoria*, and *A. tenuissima* have been acknowledged as prevalent colonizers in small-grain cereals, posing a potential risk of mycotoxin accumulation in grains across countries such as Argentina, Poland, Italy, Denmark, Norway, and Germany [12], Colque-Little et al. [7] assert the imperative of intensifying studies aimed at assessing potential hazards to quinoa production. 

Considering the significance of this phytopathogenic agent and the limited understanding of its influence on various quinoa varieties, it becomes imperative to ascertain its presence in quinoa crops, especially considering its unreported status in Colombia. This research aims to isolate species of *Alternaria* spp. from plots of quinoa present in the Boyacá department (Colombia), in order to make the molecular identification through a comparative analysis, using the molecular markers intergenic transcribed spacer (ITS) and elongation factor of translation and 1-α (*TEF-1α*) and then the pathogenicity of the isolates was determined by calculating the severity index in the Piartal quinoa cultivar under greenhouse conditions.

## 2. Materials and Methods

### 2.1. Sampling Area and Conditions

**Figure 1:**
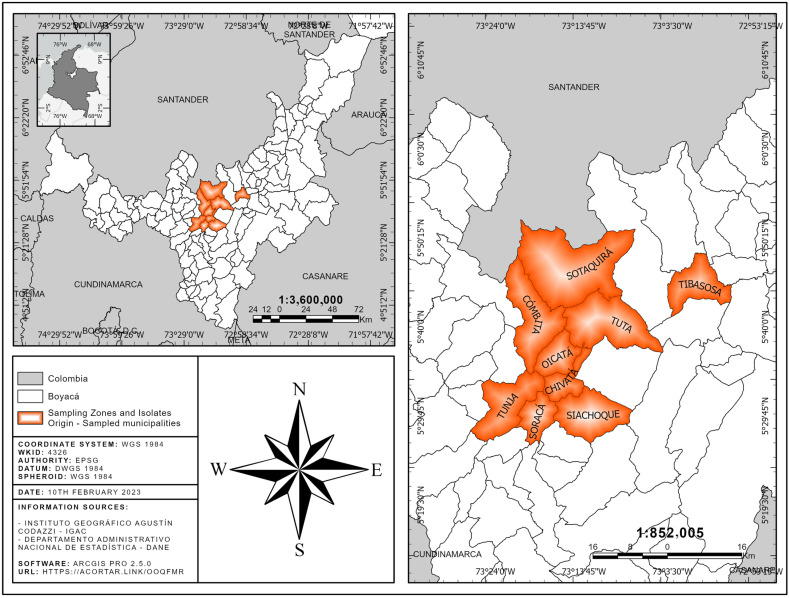
Sampling regions and isolating origin. The municipalities in which the collected leaves are shown in orange.

From twelve plots previously planted by farmers located in the department of Boyacá (Figure 1), we collected in the middle third of plants 30 to 40 samples (leaves or stems) with disease symptoms corresponding to chlorosis, necrosis, black spots, and wilting.

The plants sampled were in the ear formation, fluorescence, or maturity stages, and a maximum of 3 samples per plant were collected. The sampling was performed in eight plots located in the zones with the highest productivity (Table 1).

The plant samples collected in this investigation correspond to samples from non-wild agricultural plots; these were obtained following the relevant regulations and legislation. The sample collection permits were granted by the “Instituto de Investigación de Recursos Biológicos Alexander Von Humboldt”-Registro Único Nacional de Colecciones (RNC) of Colombia covered by the Specimens Registry in the application of article 6 of Law 1955 of 2019.

### 2.2. Isolation of Alternaria spp.

To isolate *Alternaria* spp. fungal strains from diseased leaves and stems, the collected leaves were superficially disinfected with 5% commercial sodium hypochlorite solution (Clorox brand^®^) for two minutes and 70% ethanol for five minutes; a triple wash with sterile distilled water was performed at the end of each disinfection. In order to evidence the correct disinfection of the leaves, 1 mL of the water resulting from the last wash was taken, seeded on plates with Potato Dextrose Agar (PDA) Oxoid (4 g/L potato infusion, 20 g/L glucose and 15 g/L agar, final pH 6.0+/−0.2), and incubated for seven days at 25 °C. 

To induce fungal sporulation, once disinfected, the plant segments were incubated in humid chambers corresponding to glass Petri dishes containing water agar (12%) or absorbent paper moistened with sterile distilled water and a mesh screen. The humid chambers were incubated for 7 to 15 days at room temperature for day–night light exposure until the appearance of mycelium. The mycelium was transferred to a PDA medium. The plates were incubated at 25 °C for seven days without light. Once the first mycelium was obtained from the leaves, the purification of the *Alternaria* spp. strains was performed by monosporic culture according to Pitt and Hocking [13]. For this, the spores from seven-day cultures were transferred to a petri dish containing water agar, and the individual spores were transferred to PDA agar 24 h after incubation. 

### 2.3. Morphology

Morphological characterization was made in potato-carrot agar (PCA) and V-8 juice agar V8 from monosporic cultures, and SNA medium for conidia characterization. All plates were incubated in an alternating light-dark cycle consisting of 8 h of cool-white daylight followed by 16 h of darkness at 25 °C to induce the formation of the characteristic sporulation pattern according to the Simmons [14] protocol. The cultures were observed with a stereo microscope after 5–7 days at 80× magnification. Further examination was performed at 100× magnification using a compound microscope for the conidia description lactophenol blue on a microscope slide using tape preparations. The microscopic observation of preparations stained with lactophenol blue in an Olympus Cover 015 optical microscope carried out the morphological identification by observing the characteristics of the different sporulation models and the conidia. The identification was made from monosporic cultures.

### 2.4. Alternaria Isolates Identification and Phylogeny

#### DNA Isolation

Promega’s Wizard Genomic DNA Purification Kit was used for DNA extraction modifying the plant DNA extraction protocol as described below. First, Mycelia were frozen for at least 24 h and lyophilized for 48 to 72 h with a lyophilizer Freezone Plus 4.5 L Labconco^®^. Next, 200 to 500 mg of previously lyophilized mycelium were taken, and 600 μL of nuclear lysis solution was added. The samples were incubated at 65 °C for 15 min; subsequently, 3 μL of RNase solution was added and incubated at 37 °C for 15 min and 5 min at room temperature. For protein precipitation, 200 μL of precipitating solution was added, and centrifugation was performed at 13,000× *g* for 3 min; precipitation and washing were performed according to the manufacturer’s instructions.

### 2.5. Molecular Phylogeny

The ITS intergenic regions and the elongation factor EF-1α were used as molecular markers. The primers proposed by White et al. [15] ITS1 (5′-TCCGTAGGTGAACCTGCGG-3′) and ITS4 (5′-TCCTCCGCTTATTGATATGC-3′) were used for the PCR amplification. For this, a 30 µL reaction mix contained 0.125 µM of each primer, 15 µL of 2× PCR Taq Master mix G013 abm^®^, and 1 to 1.5 µL of the template DNA sample (in a concentration range of 20 to 100 ng/µL). The reaction was performed in an Axygen^®^ MaxyGene™ thermocycler (Corner Laboratories). The initial denaturation was performed at 94 °C/3 min, followed by 30 serial cycles corresponding to denaturation at 95 °C/1 min, annealing at 56 °C/35 s, and extension at 72 °C/1 min. The final extension was performed at 72 °C/7 min.

For PCR with primers EF1-983F (5′- GCYCCYGGHCAYCGTGAYTTYAT) and EF1-2218R (5′- ATGACACCRACRGCRACRGTYTG) [16], the 30 µL reaction mix contained 1.2 µL of each primer (10 nM), 15 µL 2X PCR Taq Master mix G013 abm^®^, and 1 µL of the template DNA sample (in a concentration range of 20 to 100 ng/µL). The reaction was performed in an Axygen^®^ MaxyGene™ thermocycler (Corner Laboratories). The initial denaturation was performed at 95 °C/5 min, followed by 30 serial cycles corresponding to denaturation at 95 °C/1 min, annealing at 60.5 °C/30 s, and extension at 72 °C/1 min. The final extension was performed at 72 °C/3 min.

The sequences of two regions from the isolate’s genome were created in the Gencore DNA sequencing center (Universidad De Los Andes-Colombia). The editing and identity search of the sequences was performed with the GenIOUS Primer^®^ 2021.0.3 program. The dendrogram was constructed with the neighbor-joining statistical method, p-distance model, and bootstrap method phylogeny test of 1000 replicates with the MEGA X program. In order to verify the identified species, sequences from reference materials previously reported in the NCBI (National Center for Biotechnology Information) Gene Bank were utilized for dendrogram construction (Supplementary Materials Table S1).

### 2.6. Koch’s Postulates and Pathogenicity Test

**Figure 2:**
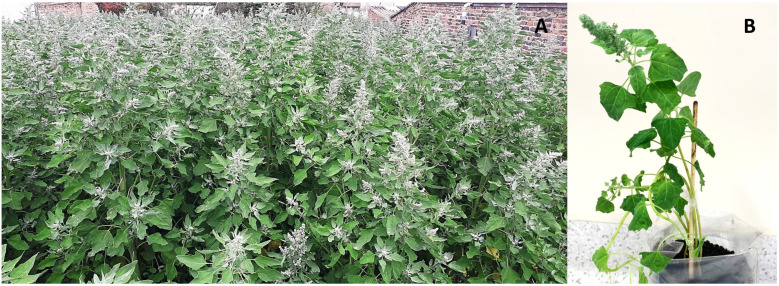
Piartal quinoa cultivar. (**A**) Plot from which the seeds were obtained. (**B**) Corresponds to the plants obtained under greenhouse conditions that were inoculated where the phenotypic characteristics of this variety are observed.

The plants used for pathogenicity tests corresponded to the Piartal variety. This variety is widely cultivated in the study area and was previously described by Morillo-Coronado et al. [11]. A farmer in the Tibasosa region supplied the seeds, which were obtained by self-pollination, guaranteeing the purity of the cultivar through strict control of cross-pollination and selection of the plant material according to the morphological characteristics of the variety. The Piartal cultivar plants were characterized by having green panicles with glomerular shape, serrated leaf margins with purple pigmentation in the axils, a green stem, and green striations (Figure 2).

The plants were grown under greenhouse conditions in 15 cm × 25 cm pots with land fertilized with cassava rice in a 3:2 ratio and irrigated every three days. Inoculation was carried out in 90-day-old plants modifying the protocol proposed by Yin et al. [5]. The isolates selected for pathogenicity testing corresponded to isolate H101 of *A. alternata* and H74, H78, and H95 of *A. scrophulariae* since these three isolates were grouped into a single clade for the two molecular markers used. Inocula were prepared from 7-day-old culture plates at a concentration of 1 × 10^5^ conidia/mL and applied by spraying. Subsequently, the plants were covered with plastic and wet paper to maintain a humid environment. The trials were conducted in two repetitions. In the first trial, the environmental conditions of the incubation corresponded to an average temperature of 22.8 °C and an average humidity of 36%, and in the second trial, the average temperature was 21 °C with a humidity of 50%. The plants were kept in day natural light conditions. Symptom development was evaluated at 7- and 15-days post-inoculation. A total of six plants per isolate were inoculated. The percentage of infected leaves on each plant was determined by taking into account the total number of leaves on each plant. The evaluation of infection severity was performed by applying the schematic scale proposed by Perina et al. [17]. In order to corroborate that the lesion observed was generated by *Alternaria*, the colonies were reisolated from the lesion of leaves employing humid chambers described before, and their identity was corroborated by colony morphology and microscopic characteristics to comply with Koch’s postulates. Tests were performed in triplicate, and statistical differences were determined by an ANOVA and the Tukey test at 5%.

## 3. Results

### 3.1. Isolation of A. alternata and A. scrophulariae

**Figure 3:**
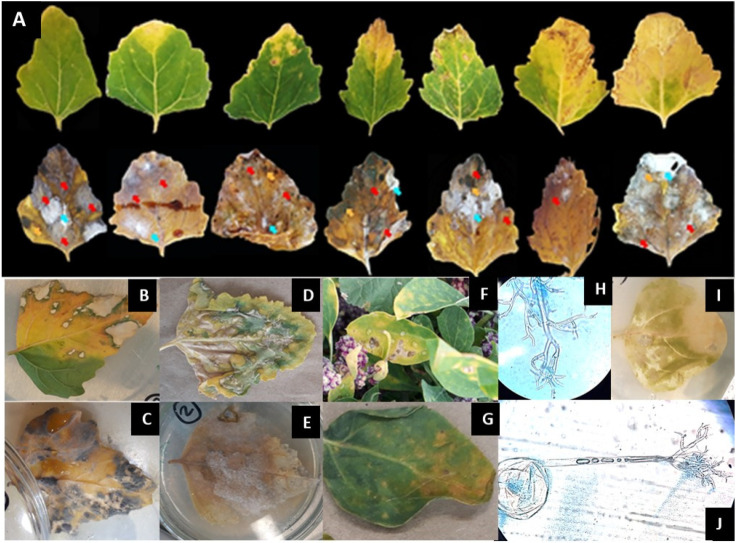
Symptomatology observed in the field. *Alternaria* was isolated from leaves with lesions (red arrow). (**A**) In the figure, the areas delimited on the leaves correspond to the areas where mycelial growth corresponding to this genus was observed; however, many leaves collected also showed the presence of *Fusarium* sp. (blue arrow) and *Cladosporium* sp. (yellow arrow). (**B**,**C**) Leaves with a high rate of *Cladosporium* sporulation. (**D**,**E**) Leaves with a high rate of *Fusarium* sporulation. (**F**,**G**) symptomatic leaves. (**H**–**J**) Leaves with the presence of *Peronospora* sp. (**J**) *Peronospora* sp. at 40× magnification.

The collected leaves showed symptoms corresponding to brownish spots with chlorotic halo, apical chlorosis, and necrosis, as well as yellowing of veins, more generalized choloris/senescent type, black necrotic spots, and apical necrosis. However, we did not find leaves that only presented *Alternaria* since this was always associated with other fungi such as *Fusarium* [14], *Cladosporium*, *Phoma,* and *Peronospora* (this was found only in the Tibasosa plot) so it is unknown if *Alternaria* induced the symptoms in the field and not another pathogen or a consortium (Figure 3). We have found *A. alternata* in Tuta, Soracá, Tunja, Siachoque, Oicatá, and *A. scrophulariae* in Tuta y Soracá. 

**Figure 4:**
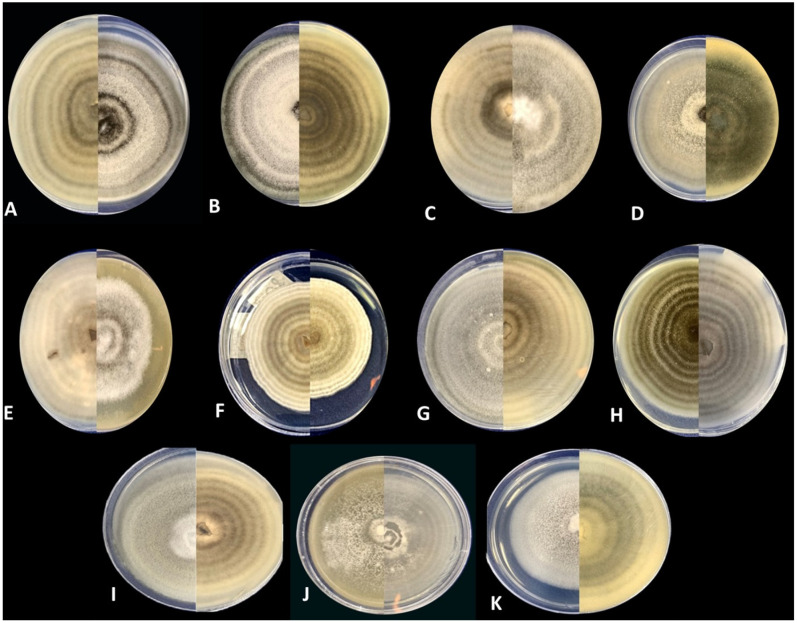
Morphology of *Alternaria* spp. *A. alternate* isolates (**A**) H49, (**B**) H61, (**C**) H67, (**D**) H73, (**E**) H75, (**F**) H97, (**G**) H100, and (**H**) H101 show colonies 4 cm in diameter, with four pairs of concentric rings. The morphotypes H87, H94, H108, and H126 presented the same macromorphological characteristics of H49, H75, H67, and H61, respectively. *A. scrophulariae* (**I**) H74, (**J**) H78, and (**K**) H95 show colonies 4 cm in diameter, with six concentric rings. The morphotypes H66 and H72 presented the same macromorphological characteristics as H78 and H66, respectively. The photographed plates correspond to the growth of seven days in PCA.

Colonies isolated and purified from monosporic cultures corresponded to *A. alternata* and *A. scrophulariae*. *A. alternata* shows a 4 cm diameter with four pairs of concentric rings of growth and sporulation (Figure 4). 

**Figure 5:**
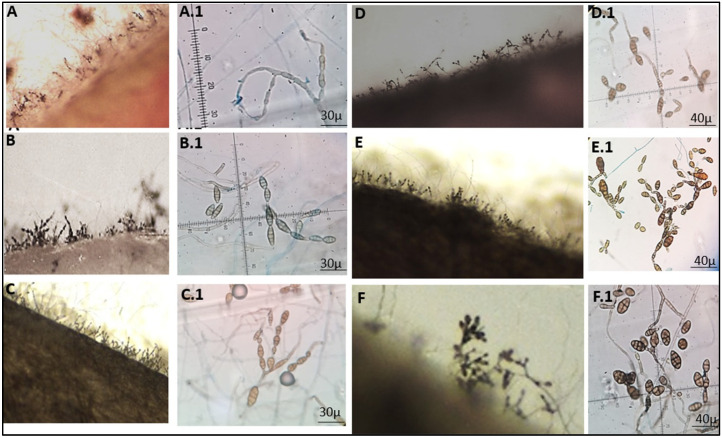
Sporulation pattern and conidies of *A. scrophulariae* H74 (**A**,**A.1**), H78 (**B**,**B.1**), and H95 (**C**,**C.1**). The sporulation pattern on a cut PCA surface is with chains of 2-3 conidia and short conodiophores. The conidia in a chain are obclavate. H66 (**D**,**D.1**), H72 (**E**,**E.1**), H101 (**F**,**F.1**) corresponding to *A. alternata*. The sporulation pattern on a cut PCA surface is abundant with chains of 4-6 conidia and short conidiophores. The first 1-2 conidia in a chain are ellipsoid or subsphaeroid. The photographed conidia correspond to the sporulation on SNA agar after seven days of growth.

The sporulation pattern is a single suberect conidiophore and an apical cluster of branching chains of small conidia separated by a short secondary conidiophore. The first 1–2 conidia in a chain usually remain long-elliptical, ovoid, ellipsoid, or subsphaeroid. The elliptical conidia are ca 26 − 30 × 5 − 9 µm (40×) with 4–7 transverse septa and a few or no longisepta. *A. scrophulariae* showed a 5 cm diameter with three pairs of concentric rings of growth and sporulation. The sporulation pattern is a single suberect conidiophore, with 2 or 3 conidia. The first 1–2 conidia in a chain usually remain obclavated. The conidia are ca 20 − 55 × 3 − 10 µm (40×) with 3–6 transverse septa and a few or no longisepta (Figure 5). 

### 3.2. Molecular Phylogeny

In the present study, 17 sequences for the ITS region and EF-1α gene were analyzed for phylogenetic tree constructions (Supplementary Materials Table S2). We found that the most predominant species corresponded to *A. alternata* with 99 to 100% similarity with the other sequences reported in the GenBank. Both markers corroborated this identity. 

**Figure 6:**
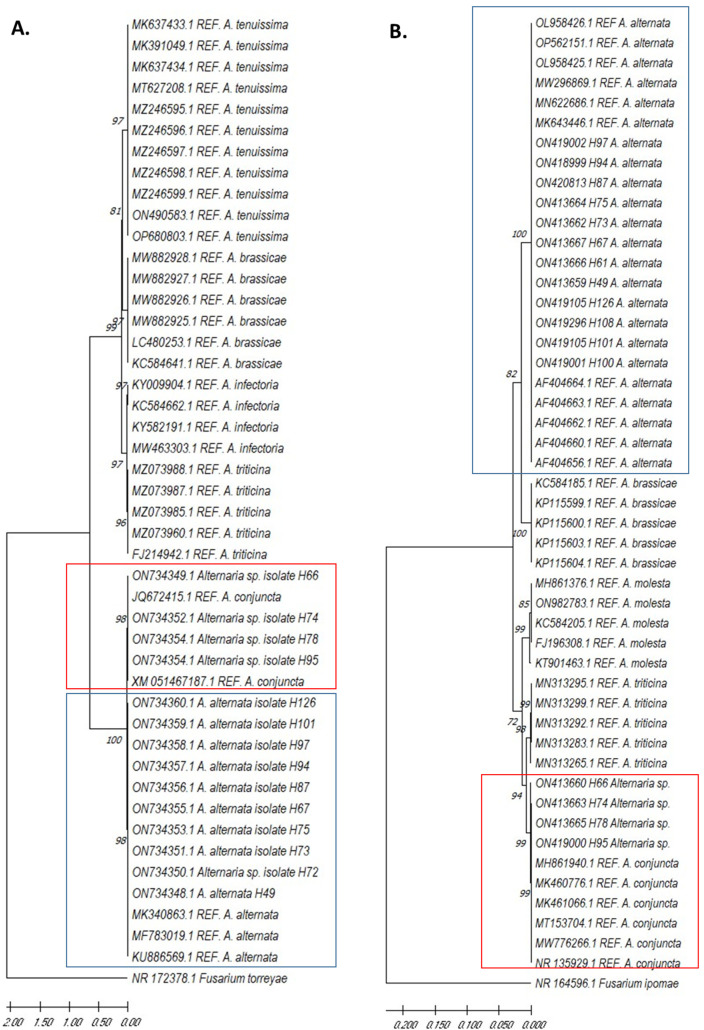
Phylogenetic tree of *A. alternata* and *A. scrophulariae* (formerly *A. conjuncta*) isolates in quinoa, based on neighbor-joining analysis of the EF1-α gene (**A**) and ITS region (**B**). Bootstrap values are from a bootstrap test of 1000 replicates. *F. torreyae* NR_172378.1 and *F. ipomoeae* NR_164596.1 correspond to the outgroup. The isolates identified as REF. correspond to sequences previously reported in the NCBI GenBank, selected as reference sequences. The blue and red boxes indicate the clustering of isolates obtained from quinoa cultures with reference sequences from the two identified species: *A. alternata* (blue) and *A. scrophulariae* (red).

We built two dendrograms with these sequences by the neighbor-joining method, both for the EF-1α gene and the ITS region (Figure 6). As an outgroup, we selected the *Fusarium torreyae* NR_172378.1) [18] and *F. ipomoeae* NR_164596.1 [19] sequences reported in GenBank. As shown in Figure 6A,B, all isolates were clearly classified into two groups, *A. alternata* and *A. scrophulariae* (formerly *A. conjuncta*), according to phylogenetic analysis.

### 3.3. Pathogenicity Test and Koch´s Postulates

**Figure 7:**
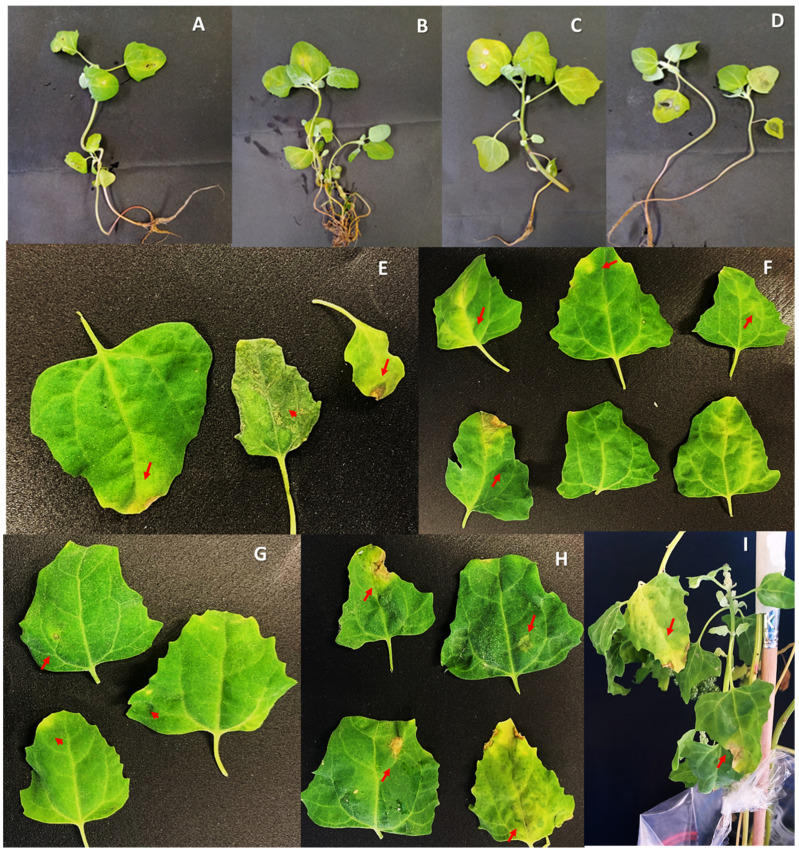
Symptomatology induced by *A. alternata* and *A. scrophulariae* on quinoa plants of Piartal variety at 10 days of inoculation. First trial (**A**) *A. alternata* and (**B**–**D**) *A. scrophulariae* Symptoms. Second trial: (**E**) corresponds to a plant inoculated with isolate H101. (**F**–**H**) correspond to symptomatic leaves obtained from plants inoculated with isolates H74, H78, and H95, respectively; (**I**) corresponds to isolate H101 of *A. alternata*. The red arrows indicate the lesions in which it was possible to rescue the mycelium of *Alternaria* spp. and corroborate its identity in order to comply with Koch’s postulates.

Under greenhouse conditions, the symptoms of the four isolates were very similar to each other, and brownish spots with chlorotic halo, apical chlorosis, and necrosis, and yellowing of the veins were also observed in the lesions (Figure 7). Therefore, we determined that under the conditions tested, all *Alternaria* spp. isolates induced some signs of disease ten days after inoculation. Adapting the severity scale according to Perina et al. [17], indices between 2 to 5 in the first trial and 2 to 4 were reached in the second trial, without finding significant differences between the isolates, according to Tukey’s test at 5% in either of two independent trials. The average percentage of leaves that presented some symptom per plant corresponded to 25% for isolate H95, 30% for isolate H74, 15% for isolate H78, and 40% for isolate H101.

## 4. Discussion

We made the first report of *A. alternata* and *A. scrophulariae* associated with quinoa crops in Colombia and demonstrated that the isolates induced necrotic and chlorotic spots under greenhouse-controlled conditions in the Piartal variety. 

In the context of isolate identification, effective differentiation among closely related species has been achieved by sequencing multiple genes and regions. These include glyceraldehyde-3-phosphate dehydrogenase (GPDH), RNA polymerase II (RPB2), the primary strain of *Alternaria* (Alt a1), TEF (translation elongation factor), and ITS (internal transcribed spacer) [11]. In the current study, we utilized the latter two genes, TEF and ITS, through a rigorous phylogenetic analysis, and two distinct groups were successfully delineated, each exhibiting well-defined clusters for *A. alternata* and *A. scrophulariae*.

Although the existence of this genus in quinoa crops has been documented in the past, there is a scarcity of comprehensive reports detailing the extent of its impact. Nonetheless, it is of paramount importance to acknowledge that the fluctuations in the occurrence of *Alternaria* spp. are linked to seasonal patterns, influencing the dispersion of pathogens and subsequently affecting disease development [20]. We successfully isolated colonies of *Alternaria* spp. from both stems and leaves. This genus has previously been reported in quinoa seeds of Argentine, Peruvian [21], and Brazilian origin [22], in the rhizosphere [23], as an endophyte, and a secondary parasite associated with high humidity. Although Dřímalková and Veverka [6] isolated *Alternaria* from infected tissue with damping-off, these isolates did not disease quinoa seedlings in pathogenicity tests. In this sense, little has been known about the pathogenic character of *Alternaria* in the quinoa crop. Of the few reports on this genus as a pathogen in this species, Yin et al. [5] reported quinoa panicle rot (QPR) caused by *Trichothecium roseum*, *A. alternata,* and *F. citri* as a new disease that represents a significant threat to this crop; interestingly, the authors report a significant difference between all three species evaluated, since *T. roseum* and *F. citri* have a higher growth and germination rate in warmer climates (25–30 °C) and humid conditions (water activity ≥0.98), while *A. alternata* preferred lower temperatures (20–25 °C). In this way, Elhadidy [24] isolated *F. solani*, *Alternaria* sp., *Acremonium* sp., and *F. oxysporum* from rotten quinoa seedlings collected in Egypt and reported that all fungal isolates were pathogenic, causing wilt and root rot. This year, Colque-Little et al. [7] published the first report of *Alternaria* spp. (*Alternaria* and *Infectoria* sections) as foliar pathogens of quinoa causing yellow leaf blotch disease, which agrees with the results obtained in this study.

Furthermore, *Alternaria* has been documented in other crops closely related to quinoa within the Amaranthaceae family. For instance, in spinach, symptoms attributable to *A. alternata* were observed on the lower and middle leaves, presenting as small circular spots with concentric rings that progressed into irregular lesions [25]. Similarly, in sugar beet, *A. tenuissima* manifested as dark brown circular or irregular spots on otherwise healthy plants. Initially, these lesions remained confined within specific parallel leaf veins [26]. In the context of amaranth, *A. alternata* emerged as one of the primary microorganisms responsible for infections and seed discoloration [27].

In our investigation, plants inoculated under controlled greenhouse conditions predominantly exhibited chlorotic spots on the tips and veins. Some sporadic necrotic spots were observed, whereas circular spots with concentric rings were infrequent and modest in size (ranging from 2 to 3 mm). This phenomenon could potentially be attributed to the relatively brief duration of the assay. However, it is noteworthy that in the case of wheat, short intervals of *Alternaria* disease induction led to higher severity and quicker symptom development under controlled inoculation conditions [28]. In Abbas et al.’s study [28], Koch’s postulates were applied to affirm the pathogenicity of the original host post-inoculation, revealing symptom emergence after several weeks. 

Additionally, our research employs Koch’s postulates to establish that isolates of *A. alternata* and *A. scrophulariae* have the capacity to induce lesions under greenhouse conditions with relative humidity ranging from 36% to 50% and an average temperature spanning 21 °C to 22.8 °C. These variables (temperature and humidity) also exert a significant influence on the severity of *Alternaria* in crops. As demonstrated by Saharan et al. [29], environmental conditions play a pivotal role, with lower temperatures and higher humidity levels enhancing the progression of *A. brassicae*. Temperature, in particular, emerges as a determining factor for the expansion and severity of leaf spots induced by *Alternaria* species. This is attributed to its influence on conidia germination, growth, penetration, and albeit subsequent colonization of the host plant.

Moreover, it is worth noting that Piartal, the quinoa variety under examination, falls within the category of “sweet” quinoa (with saponin content ≤ 0.06%) [30]. It has been established that elevated saponin levels possess the potential to impede fungal growth [31] or serve as a fungistatic agent [32]. This circumstance could have potentially contributed to the development of the disease. Consequently, it becomes imperative to incorporate these parameters into future research endeavors to gain a more comprehensive understanding the pathogenicity. 

Concerning the distribution of *Alternaria*, our investigation revealed its presence in 17 out of the 18 sampled plots. This finding contrasts with the observations made by Pappier et al. [21] in Latin America. Their study reported the occurrence of fungal species like *Alternaria*, *Cladosporium*, and *Fusarium* in quinoa crops across various locations in Argentina and Bolivia, albeit with minimal incidence. However, the notable prevalence of *Alternaria* spp. that we identified in the crops necessitates further examination, considering the extensive array of potential host plants susceptible to this pathogen and the potential economic implications that could arise as a consequence.

The significance of identifying *Alternaria* species in crops extends beyond their phytopathogenic nature, as these organisms can pose a potential risk to human health due to the production of mycotoxins. As highlighted by Botallico and Logrieco [33], species such as *A. alternata* and *A. tenuissima* are frequently linked to various diseases in small-grain cereal plants and have the capacity to generate a range of mycotoxins. Vásquez-Ocmín et al. [34] conducted studies detecting several mycotoxigenic fungi, including *Aspergillus* sp., *Penicillium* sp., and *Alternaria* sp., and correlated the presence of these fungi with the occurrence of mycotoxins in pseudocereals.

While our study did not encompass an assessment of the toxicogenic potential of the isolates, it is crucial to acknowledge that the genus *Alternaria* carries significant implications for food quality due to its propensity for mycotoxin production. Notably, species within the *Alternaria* genus are known to synthesize more than 70 toxic secondary metabolites that affect plants (phytotoxins). Hence, it is recommended that forthcoming research endeavors focus on evaluating the quality of flour and grains obtained from quinoa that has been impacted by this pathogen, particularly considering its widespread presence across the majority of the sampled plots.

Finally, we recommend the initiation of comprehensive studies encompassing both field and controlled environments to assess the impact of *Alternaria* spp. on quinoa crops. Such studies should consider the varietal differences and the influence of environmental conditions. It is advisable to employ models that can shed light on significant aspects, similar to those observed in cases of mildew. These aspects encompass the substantial variation evident in infection severity, sporulation rates, and incidence rates. Additionally, it is worth exploring factors that exhibit minimal variation in their correlation with mildew tolerance, such as the altitude of the origin site and the saponin content of the seed. Furthermore, an investigation into the relationship between the width of stomata and infection severity could provide valuable insights into this complex interaction [32]. Conducting such holistic research will contribute to a more nuanced understanding of the dynamics between *Alternaria* and quinoa, encompassing its diversity, adaptability, and potential impact on crop yield and quality. Our research conducted in the department of Boyacá, Colombia, has identified a substantial prevalence of *Alternaria* spp. within quinoa crops. While we managed to replicate certain symptoms in controlled greenhouse conditions, it remains imperative to establish extensive trials aimed at assessing the repercussions of *Alternaria* infection and its intricate interplay with the various quinoa varieties cultivated in the field.

We have identified *A. alternata* and *A. scrophularia* as two distinct phylogenetic clusters. Expanding the repertoire of molecular markers is recommended to further elucidate the quinoa-related species, allowing us to ascertain whether different pathovars contribute to varying degrees of infection. However, noteworthy differences were not observed among the tested isolates in our pathogenicity assessments.

The study of this genus holds paramount importance not only due to its role as a phytopathogen but also due to its mycotoxigenic nature, which poses potential health risks associated with the consumption of derived food products. As we advance, it is imperative to continue exploring the diverse aspects of *Alternaria*’s interaction with quinoa, encompassing its ecology, genetic diversity, and impact on both crop production and human health.

## 5. Conclusions

In this research, we have identified two phytopathogenic *Alternaria* species associated with quinoa cultivation for the first time in Colombian crops. Both *A. alternata* from the *Alternaria* section and *A. scrophulariae* from the *Infectoriae* section have been reported as foliar fungi causing significant losses in various crops. However, their pathogenic effect on quinoa is poorly understood. Through pathogenicity assays under greenhouse conditions, we have determined that the Piartal variety is susceptible to foliar damage caused by these two species, with no significant differences observed in the degree of pathogenicity. The impact of these two species on quinoa production and the quality of its derivatives remains to be elucidated. This consideration should encompass not only their phytopathogenic characteristics but also their mycotoxigenic potential.

## Figures and Tables

**Table 1:** Characteristics of sampling regions for the *Alternaria* isolates obtained.

Town	Location	High m.a.s.l	Average T °C	Sampled Varieties	Plot Extention, Ha
Tunja	Porvenir I	3080	14	Blanca de Jericó	1.2
	Porvenir II	3060	11	Blanca de Jericó	1
Cómbita	San Onofre	2764	13.5	Blanca de Jericó	1.5
Cómbita	San Martín	2762	13.5	Blanca de Jericó	0.7
Siachoque	Tocavita	2778	11.2	Blanca Jericó	1.2
	Guatichá	2778	12	Piartal	1.5
Tuta	Hacienda	2721	11	Blanca de Jericó	1.2
	Agua Blanca	2710	13.5	Blanca de Jericó	1.5
Soracá	Otro lado	2710	12	Blanca de Jericó	0.9
Tibasosa	Peña Negra	2535	12.8	Piartal	1
Chivata	San Francisco	2657	11	Blanca de Jericó	1
Sotaquirá	Cortadero	2760	11	Piartal	0.9

## Data Availability

All sequences produced in this study are publicly available in the NCBI GenBank Database, https://www.ncbi.nlm.nih.gov// accessed on 1 August 2023 (accession numbers are registered in Supplementary Material Table S2). Fungal isolates are available in the “Colección de Hongos y Microorganismos de la Universidad de Boyacá”, UBCHM.

## Supplementary Materials

The following supporting information can be downloaded at: https://www.mdpi.com/article/10.3390/jof9090947/s1, Table S1: Reference sequence used for dendrograms construction; Table S2: List of *A. alternata* and *Alternaria* spp. isolates sequenced in the present study and their GenBank information together with their relatives to build a phylogenetic.
